# How Cell Number and Cellular Properties of Blood-Banked Red Blood Cells of Different Cell Ages Decline during Storage

**DOI:** 10.1371/journal.pone.0105692

**Published:** 2014-08-28

**Authors:** Wei-Wei Tuo, Di Wang, Wen-Jing Liang, Yao-Xiong Huang

**Affiliations:** Department of Biomedical Engineering, Ji Nan University, Guang Zhou, China; Emory University/Georgia Insititute of Technology, United States of America

## Abstract

**Aims:**

Numerous studies have suggested that transfusion of red blood cells (RBCs) stored over a long period of time may induce harmful effects due to storage-induced lesions. However, the underlying mechanisms responsible for this damage have not been identified. Furthermore, it is unclear why and how up to 30% of long-stored RBCs disappear from the circulation within 24 hours after transfusion. The aim of this study was to determine how the cell number of RBCs of different ages changes during storage and how these cells undergo cumulative structural and functional changes with storage time.

**Methods and Results:**

We used Percoll centrifugation to fractionate the RBCs in blood bank stored RBC units into different aged sub-populations and then measured the number of intact cells in each sub-population as well the cells’ biomechanical and biochemical parameters as functions of the storage period. We found that the RBC units stored for ≤ 14 days could be separated into four fractions: the top or young cell fraction, two middle fractions, and the lower or old fraction. However, after 14 days of storage, the cell number and cellular properties declined rapidly whereby the units stored for 21 days only exhibited the three lower fractions and not the young fraction. The cell number within a unit stored for 21 days decreased by 23% compared to a fresh unit and the cells that were lost had hemolyzed into harmful membrane fragments, microparticles, and free hemoglobin. All remaining cells exhibited cellular properties similar to those of senescent cells.

**Conclusion:**

In RBC units stored for greater than 14 days, there were fewer intact cells with no healthy cells present, as well as harmful membrane fragments, microparticles, and free hemoglobin. Therefore, transfusion of these stored units would not likely help patients and may induce a series of clinical problems.

## Introduction

Red blood cell (RBC) storage lesion has recently been recognized as an important issue facing transfusion medicine [1]. The issue has attracted numerous studies to determine the potential risks associated with transfusion of RBCs stored over a longer period of time and the underlying mechanisms responsible [2]–[7]. Several major projects are ongoing [8], [9], and clinical trials and laboratory studies have already shown that long-stored red blood cells have harmful effects [4], [9]–[19]. The structural and biochemical changes that RBCs go through during storage are likely to contribute to adverse transfusion effects [3], [11], [19]–[25]. A definitive determination of the potential risks associated with transfusion of RBCs stored for longer periods of time, however, is still elusive not only because the responsible mechanisms have not yet been identified, but also because some facts are not clear. For example, it is unknown why and how up to 30% of long-stored RBCs rapidly disappear from circulation within 24 hours after transfusion [26]. The number of intact RBCs that actually remain in a long-stored RBC unit before transfusion is also unknown and merits further research.

A human RBC has a lifespan of approximately 120 days. Under normal circumstances, approximately 2.4 million new RBCs are produced per second with the concomitant removal of a similar number of senescent RBCs from the circulation. Therefore, human blood contains RBCs that range from 0 to 120 days of age, which is equivalent to a unit of freshly drawn RBCs. Young RBCs can survive for a long period of time after transfusion, but senescent RBCs are rapidly eliminated from the circulation. Therefore, to evaluate the survival time of blood-banked RBCs after transfusion, it is important to determine the proportions of young and old RBCs in the blood-banked RBC unit as well as assess how the proportions and the cells’ properties change during storage. To obtain this information, fractionation of RBCs into subpopulations based on cell age is required. There are various methods for fractionating RBCs based on age [27]–[29], and among them Percoll gradient centrifugation is a simple yet effective approach. In our previous study [30], we found that freshly drawn human blood can be fractionated by Percoll gradient into four subpopulations. The topmost and lightest layer contains the “young (Y)” RBCs; the M1 and M2 middle layers; and the bottom and densest layer contains the “old (O)” RBCs. Moreover, the biomechanical and biochemical properties of the cells decline in order of fractions Y, M1, M2, and O, indicating that the four RBC sub-populations have different cell ages and viabilities. The RBCs in the Y fraction have a high electrical charge density (zeta-potential) so they repel high charge-bearing monocyte subsets that give rise to equally highly charged subsets of macrophages, and have good deformability for migrating through capillaries in circulation. However, the zeta-potential of RBCs in the O fraction (–23.2 mV) was found to decrease by approximately 30% compared to the Y-RBCs due to a decrease in sialic acid. The cells’ membrane deformability also collinearly decreased to a level at which the cells would be too stiff to pass through narrow capillaries in the spleen and therefore would become trapped and easily eliminated by the reticuloendothelial system (RES). Resultantly, it was estimated that RBCs in the O fraction do not survive longer than 32 days in circulation [31]; however, it is not clear if this is the case for RBCs in blood-bank stored RBC units. Furthermore, it is unclear how the number and cellular properties of RBCs in each age fraction vary with storage time and whether the cells would age quicker under blood bank storage conditions, thus resulting in a shorter survival time.

In this study we used Percoll gradient centrifugation to fractionate RBCs from blood bank stored RBC units into different age subpopulations and performed a systematic analysis on the number of intact RBCs in each age fraction as well as the structural and functional parameters of the cells as a function of storage time. The parameters assessed include the cell’s morphological and rheological properties, their surface charge, ATP enzymatic activity, and 2, 3 diphosphoglycerate (DPG) level. Based on this study, we aimed to also obtain detailed information about how the cell number in different fractions declines as a function of storage period. Thereby, we can obtain valuable evidence that will increase the understanding of RBC storage lesion, and help to predict the cell fate after transfusion and to more accurately evaluate the risk of transfusing the long-stored RBC units into circulation.

## Materials and Methods

The protocol used in this study was approved by the Ji Nan University Animal Care and Use Committee and conforms to the Chinese Public Health Service Police on Human Care and Use of Laboratory Animals.

### Sample collection

Eight bags of leuko-reduced CPDA-1 RBC units stored under standard blood-bank conditions were obtained from the Guangzhou Blood Services Center, China. RBC samples were prepared from the RBC units stored for different time periods (4 days, 7 days, 14 days, or 21 days) and designated as Unit 4, Unit 7, Unit 14, and Unit 21, respectively.

### Discontinuous percoll density gradient centrifugation

For every storage period, 5 ml of the RBC suspension were taken from each RBC unit and randomly divided into five 1 ml parallel samples. Each of the 40 parallel samples (diluted with buffer 1∶1) were centrifuged at 2700×g for 20 min over Percoll gradients (Pharmacia, Sweden) as previously described [30]. The gradient was constructed in five layers of 2 mL each containing 1.0636 g/mL, 1.0679 g/mL, 1.0702 g/mL, 1.0769 g/mL, and 1.0814 g/mL Percoll, respectively, buffered with HEPES containing bovine serum albumin (BSA). The composition of the BSA-buffer was as follows: 26.3 g/L bovine serum albumin, 137 mmol/L NaCl, 4.6 mmol/L KCl, and 10 mmol/L HEPES, pH 7.4. Four fractions were obtained after the centrifugation of Unit 4, Unit 7, and Unit 14, whereas Unit 21 only exhibited three fractions. Cells harvested from each fraction were washed twice in PBS and kept at 4°C for subsequent analyses. All of the cell suspensions were prepared at room temperature and their pH values were also measured as a function of the storage period.

### Cell counting

The number and proportion of RBCs in each fraction were determined by standard methods using a hemacytometer. During measurements, 10 µL of diluted RBC suspension taken from each fraction was pipetted into the hemacytometer for cell counting. At least 5 parallel samples per fraction were counted to determine the average cell number per unit volume in the fraction, which was then multiplied by the volume of the fraction to calculate the total cell number.

### Measurement of morphology

A multi-dimensional microscope was used to observe and measure the morphological parameters, including contact area and roundness of single living RBCs as previously described [32]. At least 100 cells per sample were analyzed for this measurement.

### Measurement of membrane deformability

Membrane bending modulus K_c_ was measured by using the dynamic imaging and analyzing method as previously reported [33] to determine RBC deformability. At least 30 cells per sample were analyzed for the measurement.

### Measurement of zeta-potential

A Phase-Shift-Based Analysis Light Scattering (PALS) potential-analyzing instrument (Brookhaven, New York, USA) was used to determine the zeta-potentials of red blood cells suspended in 0.029 M NaCl/4% glucose (5–8×10^6^ cells/ml) (25°C) [34], [35]. Five parallel samples were measured for each cell suspension.

### Measurement of ATP enzymatic activity

The ATP enzymatic activities, including both Na^+^ and K^+^-ATP enzymatic activities of the RBCs stored for different periods, were determined by using an ultra-micro ATPase assay kit (Jiancheng Bioengineering Institute, Nanjing, China).

### Measurement of 2, 3-DPG levels

The 2,3-DPG levels of RBCs stored for different periods were determined by ELISA. The ELISA kits used to test the blood cytolysate samples from each fraction were from EIAab Science Company, Wuhan, China.

### Measurement of membrane fragments and microparticles

The supernatant in the RBC suspension stored for 21 days was analyzed with a phase contrast microscope (Nikon TE 300, Japan) and measured using a dynamic light scattering technique on a Zeta PULS instrument (Brookhaven, NY, USA). This approach was used to detect the existence and size distribution of membrane fragments, microparticles, and free hemoglobin.

### Measurement of pH values

The pH values of the RBC units stored for different periods were measured using a FE20 pH Sensor from the METTLER-TOLEDO Company, China.

### Statistical analysis

Statistical analyses using a Student’s t-test were performed using the SPSS 15.0 statistical software. For the significant differences level examination, the *p* values were obtained by making a comparison between the cells from different fractions of all the units and the cells in the Y fraction of Unit 4; * indicates *p*<0.05 and ** indicates *p*<0.01. The *p* values were also calculated to determine if there were significant differences between the cells of each fraction from two successive storage periods; † indicates *p*<0.05 and ‡ indicates *p*<0.01.

## Results

**Figure 1:**
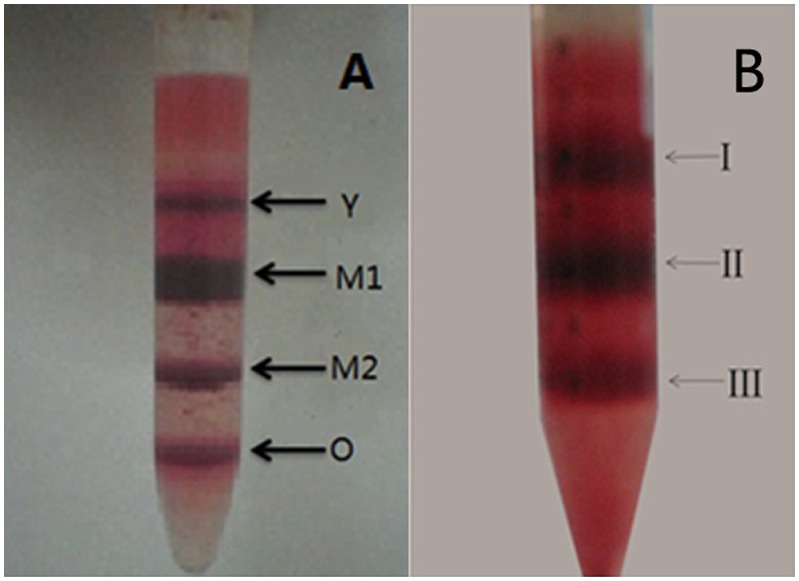
Representative images of Percoll-centrifuged samples. (A) Units stored for ≤ 14 days, (B) units stored for 21 days.

**Figure 2:**
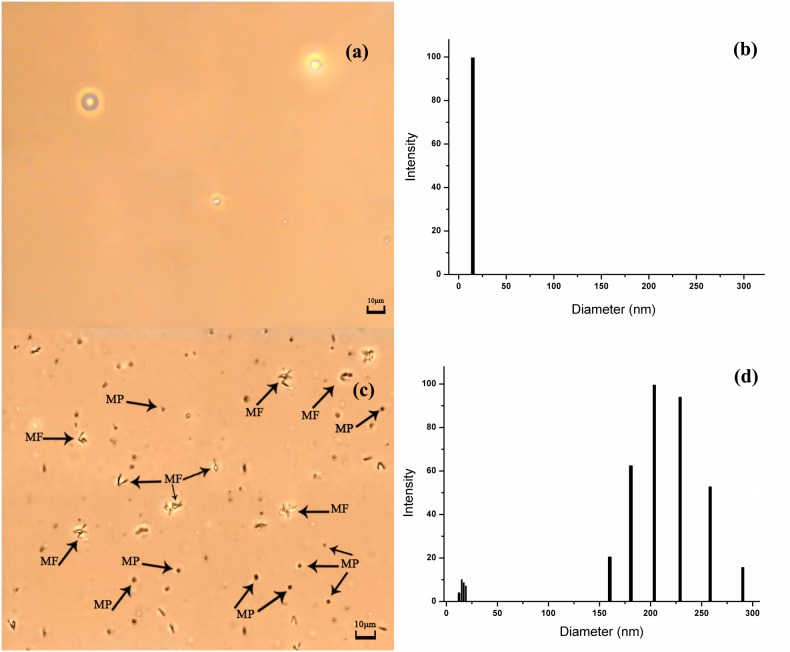
The results of measurements on the Unit 4 and Unit 21 supernatants. (a) A typical image of the supernatant of Unit 4 under phase contrast microscope. (b) A typical result of dynamic light scattering measurement on the supernatant of Unit 4. (c) The image of the supernatant of Unit 21 whereby some membrane fragments (MF) and microparticles (MP) are indicated. (d) A typical result of dynamic light scattering measurement on the supernatant of Unit 21. The histogram indicates that there were two groups of particles in the supernatant.

Images of the Percoll-centrifuged samples are shown in Figure 1. We found that the samples from units stored for 4, 7, and 14 days (Unit 4, Unit 7, and Unit 14, respectively) were able to be separated into four fractions (Y, M_1_, M_2,_ and O) by Percoll gradient as previously reported [30], whereas the samples stored for 21 days could only be separated into three fractions. Since it was not clear what kind of cells were in the three fractions, we temporarily labeled them as fractions I, II, and III, respectively (see Figure 1). In contrast to the clear and distinct boundaries between each of the four fractions in the samples of Unit 4, Unit 7 and Unit 14, the boundaries between each of the three fractions in the samples of Unit 21 were not clear. According to the principles of Percoll density gradient centrifugation and our previous study [35]–[37], the age of cells in the upper fraction was younger than that of cells in the lower fraction. This was confirmed by the biomechanical and biochemical properties of the cells determined in each fraction. The age of the cells in the four fractions was in the order of Y < M_1_< M_2_< O (or I < II < III in Unit 21). Contrary to the clear supernatant in the RBC unit stored for ≤ 14 days, the supernatant in the RBC unit stored for 21 days after Percoll gradient centrifugation was quite turbid and red, indicating that hemolysis had occurred in the unit. Figure 2 shows the images observed under a phase contrast microscope and the result of dynamic light scattering measurements on five parallel samples of the supernatants. As shown in Figure 2, the supernatant of Unit 4 was quite clear under microscope (Fig. 2a) and laser light scattering measurement only detected some particles with sizes of about 14.9 nm (Fig. 2b). According to the results of our previous measurement on hemoglobin [38], these particles were hypothesized to be aggregates of free hemoglobin. However in the supernatant of Unit 21, many membrane fragments (MF) and microparticles (MP) with sizes ranging from 0.96 µm to 2.8 µm were observed under microscope (Fig. 2c). In addition, there were also two groups of particles with sizes in the nanometer range present in the supernatant of Unit 21 (Fig. 2d). Besides the aggregates of free hemoglobin with sizes ranging from 13 to 19 nm, there were some particles with sizes ranging from 160 to 291 nm. Based on previous reports [39], [40], we concluded these were microparticles.

Table 1 lists the pH values and the number of cells in each fraction of the samples stored for different periods. The results indicated that in the RBC units stored for ≤ 7 days, approximately 60% of the cells were located in the two middle fractions in the following order based on cell number: M_1_ > M_2_ > Y > O. However, in the RBC units stored for 14 days, the order was M_1_ > M_2_ > O >Y. In the RBC units stored for 21 days, the order was I > II > III. These results indicate that the cell number in the Y and O fractions increased during the first week of storage but decreased in the M_1_ and M_2_ fractions. The increase in the number of cells in the O fraction was due to the aging of cells, whereby some RBCs that were originally in the M2 fraction had aged. However, the increase in cell number in the Y fraction during the first week of storage could be attributed to the effect of the CPDA-1 preservation solution, which improved the viability of some RBCs originally in the M1 fraction of Unit 4 so that the cells moved up to the Y fraction. After 14 days of storage, however, the cell number of the Y fraction decreased rapidly. By the third week, only three fractions (I, II, and III) remained in which the cell numbers of these fractions were equivalent to those of the M_1_, M_2,_ and O fractions in the unit stored for 14 days, respectively.

**Table 1:** The pH value and number of red cells (×10^9^) in each fraction of samples with various storage periods.

	4 Days	7 Days	14 Days		21 Days
Y	0.89±0.03	0.98±0.05^†^	0.72±0.09^†^		
M_1_	1.43±0.07	1.29±0.08^†^	1.29±0.10	I	1.25±0.13
M_2_	1.18±0.08	1.17±0.09	1.20±0.10	II	1.07±0.16
O	0.67±0.10	0.72±0.09	0.96±0.10^†^	III	0.88±0.16
Total numberpH value	4.17±0.047.11±0.05	4.16±0.047.01±0.04	4.17±0.086.92±0.07		3.20±0.13^‡^6.53±0.09

(^†^ *p*<0.05, ^‡^ *p*<0.01).

**Figure 3:**
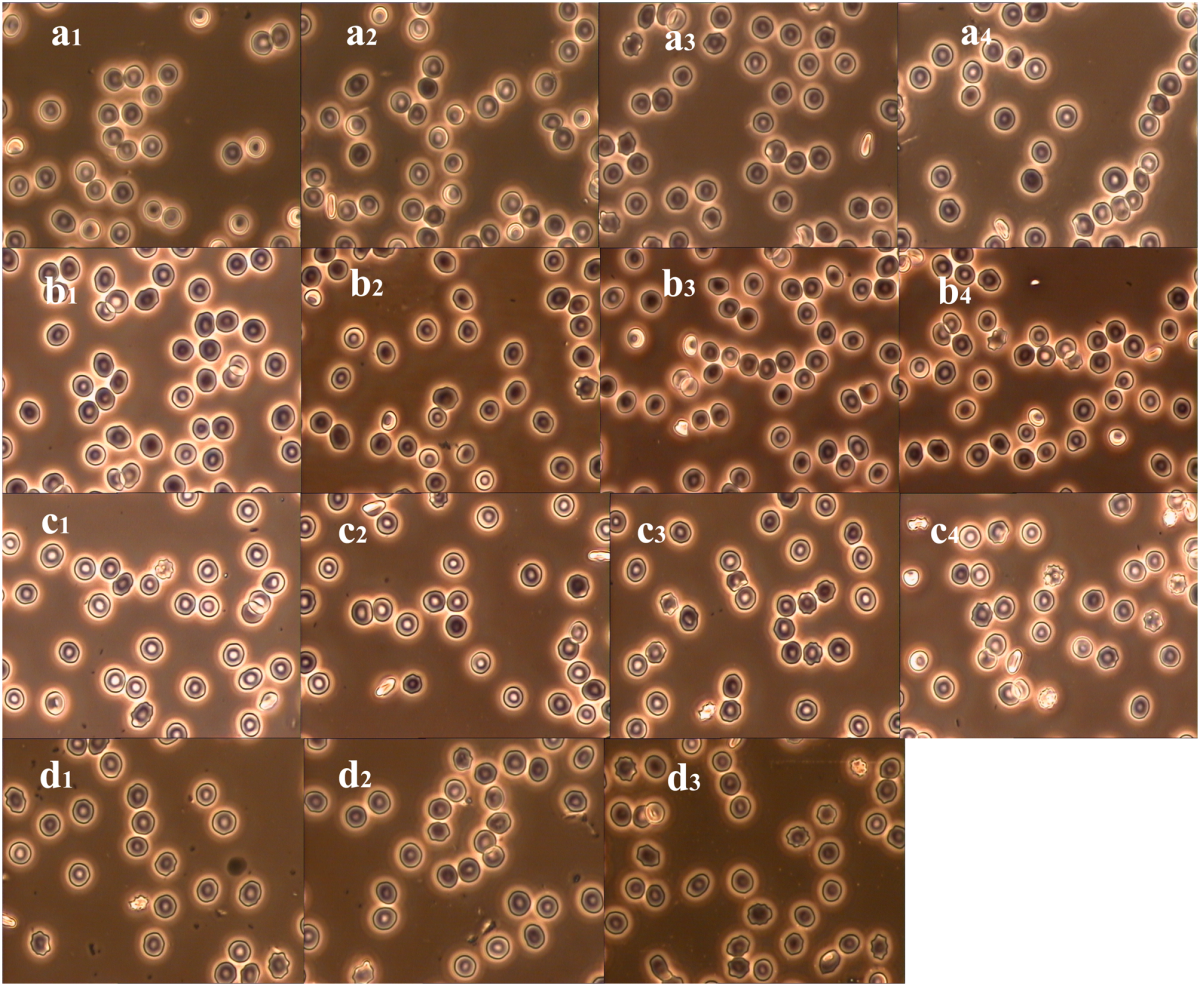
The morphology of the RBCs in each fraction at different storage periods. a_1_, a_2_, a_3,_ and a_4_ are the RBCs of Y, M1, M2, and O fractions in Unit 4_,_ respectively; b_1_, b_2_, b_3,_ and b_4_ are the RBCs of Y, M1, M2, and O fractions in Unit 7_,_ respectively; c_1_, c_2_, c_3,_ and c_4_ are the RBCs of Y, M1, M2, and O fractions in Unit 14_,_ respectively; and d_1_, d_2_, and d_3_ are the RBCs of I, II, and III fractions in Unit 21_,_ respectively.

Table 2 shows the morphological parameters of the cells in each fraction of the samples from various storage periods. The parameters include the contact area and the regular form factor (RFF), which is defined as the extent to which a structure differs from a sphere. The results clearly indicate that the older cells had a smaller contact area (also observed in Figure 3). Notably, the contact area of the old cells in the samples stored for longer than 14 days was approximately 16% smaller than that of the Y cells stored for 4 days, which indicates an obvious reduction of cell size during aging. In contrast, the RFF value increased with cell age. RFF values closer to 1 indicate a cell shape that is more likely to be spherical. Therefore, these results indicate that more cells become spherocytes or stomatocytes as the storage time increases. This was confirmed by the morphology of the RBCs shown in Figure 3 for each fraction from the different storage periods. In the Y and M1 fractions from the units stored for 4 days and 7 days (see Fig. 3a1, a2, b1, and b2), the RBCs had a typical biconcave and discoid shape; however, in the M2 and O fractions, some cells exhibited uneven edges and various membrane protrusions or even showed thorn-like projections on the surface (see Fig. 3a3, a4, b3, and b4). Several cells became stomatocytes and echinocytes, especially in the unit stored for 21 days (Fig. 3d2 and d3).

**Table 2:** Morphological parameters of the cells in each fraction of the samples under various storage periods.

	4 Days	7 Days	14 Days		21 Days
Contact Area (µm^2^)					
Y	46.42±4.61	45.64±4.55	42.68±5.00^*^		
M_1_	45.88±5.07	43.42±4.30^*^	41.32±4.91^*^	I	43.14±4.50^*^
M_2_	44.26±4.71	42.26±4.39^*^	39.84±4.62^**^	II	41.08±4.16^*^
O	43.14±4.57^*^	41.08±4.61^*^	38.87±5.09^**^	III	38.80±4.62^**^
RFF					
Y	0.64±0.05	0.69±0.06	0.74±0.06^*^		
M_1_	0.68±0.05	0.72±0.06	0.78±0.06^*^	I	0.79±0.06^*^
M_2_	0.73±0.06	0.76±0.06^*^	0.81±0.07^*^	II	0.83±0.06^**^
O	0.77±0.07^*^	0.79±0.07^*^	0.84±0.07^**^	III	0.85±0.05^**^

(*p<0.05, **p<0.01).

Table 3 provides the K_c_ value and zeta potential of the cells in each fraction of the samples stored for various periods and shows that K_c_ increased with cell age. The K_c_ value of the cells in the III fraction from unit 21 even increased 108% more than the Y fraction in the sample from unit 4. Therefore, older RBCs have a diminished ability to deform because this characteristic is inversely proportional to the K_c_ value. The deformability of the cells in all of the fractions decreased quickly as a function of time, especially between the 7th and 14th day of storage (*p*<0.01).

**Table 3:** The K_c_ value and zeta potential of the cells in each fraction of the samples under various storage periods.

	4 Days	7 Days	14 Days		21 Days
K_c_ Value (×10^9^ J)					
Y	1.586±0.013	1.713±0.015^*†^	2.187±0.031^**‡^		
M_1_	1.712±0.015^*^	1.876±0.019^*†^	2.405±0.028^**‡^	I	2.625±0.086^**†^
M_2_	2.000±0.028^**^	2.217±0.018^**†^	2.704±0.008^**‡^	II	3.018±0.140^**†^
O	2.199±0.034^**^	2.409±0.012^**†^	2.861±0.009^**‡^	III	3.298±0.179^**†^
ζ-potential (mV)					
Y	−28.05±1.07	−24.60±1.00^*‡^	−21.97±1.18^*†^		
M_1_	−28.01±1.02	−23.47±2.11^*‡^	−20.90±3.41^*†^	I	−19.83±1.13^**^
M_2_	−27.21±2.43	−21.04±2.84^*‡^	−19.95±3.05^*^	II	16.58±2.32^**^
O	−24.80±1.20^*^	−19.78±0.93^**‡^	−16.94±0.85^**†^	III	−14.81±0.75^**†^

(*p<0.05, **p<0.01; ^†^ *p*<0.05, ^‡^ *p*<0.01).

The zeta-potential also decreased as a function of cell age and storage time, particularly during the first week of storage (Table 3). Compared to the Y cells stored for 4 days, the zeta-potential of the O cells stored for 14 days decreased by 39.6%, and the cells in the III fraction stored for 21 days decreased by 47.2%.

Na^+^/K^+^-ATPase plays an important role in maintaining the ATP supply for the metabolic activity of the sodium/potassium pump. The activity of Na^+^/K^+^-ATPase is a known indicator that reflects the physiological status of RBCs. Table 4 shows the Na^+^/K^+^-ATPase activity of cells in each fraction of the samples from various storage periods. The results showed that the Na^+^/K^+^-ATPase activity began to decrease during the first week of storage and reduced abruptly during the second week (*p*<0.01). The cells in all fractions of the units stored for 14 days had Na^+^/K^+^-ATPase activity less than 50% of the values of the cells in the units stored for 4 days and approximately 40% less than that of the RBCs in unit 7. For the RBCs in unit 21, the Na^+^/K^+^-ATPase activity was even lower.

**Table 4:** The Na^+^, K^+^-ATPase activity and 2, 3-DPG level of cells in each fraction of the samples under various storage times.

	4 Days	7 Days	14 Days		21 Days
Na^+^, K^+^-ATPase activity (U/gHb)					
Y	26.871±3.960	22.883±3.558	11.970±1.579^**‡^		
M	27.004±3.450	21.450±2.985^*†^	11.248±0.746^**‡^	I	10.345±0.810^**^
M	25.364±4.381	21.777±2.939^*^	11.078±0.816^**‡^	II	8.864±0.570^**†^
O	23.587±4.717^*^	20.879±1.571^*^	10.740±0.972^**‡^	III	8.319±0.405^**†^
2, 3-DPG level (nmol/ml)					
Y	2.643±0.212	2.036±0.039^*‡^	1.996±0.043^**^		
M	2.284±0.111^*^	1.996±0.059^**†^	1.964±0.034^**^	I	1.839±0.050^**†^
M	2.248±0.094^*^	1.966±0.061^**†^	1.931±0.038^**^	II	1.714±0.043^**‡^
O	2.121±0.049^*^	1.972±0.075^**†^	1.968±0.030^**^	III	1.709±0.040^**‡^

(*p<0.05, **p<0.01; ^†^ *p*<0.05, ^‡^ *p*<0.01).

Table 4 also shows that the 2, 3-DPG levels decreased gradually as a function of storage time, which was similar to previously reported results [41], [42]. The 2, 3-DPG levels in the cells from Unit 21 were approximately 80% of the older RBCs in Unit 4. Since the 2, 3-DPG levels in RBCs reflect the affinity of hemoglobin for O_2_, these results suggest that the oxygen delivery capacity of aged cells declines with storage time. Importantly this finding is consistent with our previous results using Raman spectroscopy to assess the affinity of hemoglobin for O_2_ [30].

## Discussion

In this study we found that RBC units stored for 21 days unexpectedly exhibited only three separated fractions, and approximately 23% (∼0.96–0.98×10^9^) of the total number of cells had been lost. The age of the RBCs likely accelerated during the 21 days of storage and thus the cells originally in the O fraction of unit 14 had hemolyzed. This hypothesis is supported by the fact that the number of lost cells (∼0.96–0.98×10^9^) was the same as the number of old cells present in the O fraction of unit 14 (0.96×10^9^). Furthermore, according to the density position of the fractions in the Percoll gradients, the I, II, and III fractions from the RBC unit stored for 21 days were equivalent to the M_1_, M_2,_ and O fractions in the RBC units stored for ≤ 14 days, respectively. This was confirmed by the morphological and rheological properties as well as the surface charges, Na^+^/K^+^-ATPase activity, and 2, 3-DPG levels in the cells. Based on the capillary aspiration experiments in our previous study [35], the RBCs with a K_c_ value greater than 3×10^9^ J and a zeta potential magnitude less than −17 mV would likely be too rigid to negotiate narrow capillaries in the spleen and may become trapped and subsequently eliminated by the RES. Moreover, as their electrostatic barrier was weakened and overcome by the awaiting monocytes/macrophages, they would be easily recognized via the Fc region, undergo phagocytosis, and be cleared by the RES. The low Na^+^/K^+^-ATPase activity and 2, 3-DPG level also indicate that the cells had lost most of their energy and oxygen releasing ability. Therefore, although the RBCs in the II and III fractions of Unit 21 were still intact, they had already aged and become senescent cells on the verge of death, which would be easily eliminated by the RES shortly after a blood transfusion. The observation that 30% of the red blood cells disappeared within 24 h post-transfusion when units older than 14 days were used is likely due to the 23% loss of cells in these units and the fact that most of the remaining cells were on the verge of death.

The problems and risks of transfusing RBC units stored for 21 days into the circulation are quite clear. Not only did these units have fewer intact cells than the fresher units, but the remaining cells were also likely unhealthy, senescent, and on the verge of death. Many of the cells had hemolyzed into a large amount of membrane fragments, microparticles, and free hemoglobin as well as some haemolytic complements in the suspension [43], [44]. Since these substances have been proven to be harmful and can cause side effects such as fatal bleeding disorders, renal failure, and cardiovascular dysfunction if they are transfused into circulation [45], [46], transfusing these RBC units into circulation may induce severe adverse effects.

Tables 2–4 provide detailed information on the cell ageing process in blood stored under standard conditions of blood bank. Storage lesions began during the first week of storage and accelerated during the second week. At the end of the second week the structural and functional parameters of all cells were clearly worse than that of the old cells in the units stored for 4 days. By the third week, all of the cellular properties quickly deteriorated to the level of senescent cells. Based on previous research [31], [47], the mean survival time of RBCs in the uppermost fraction of a fresh RBC unit is approximately 88 days, whereas the RBCs in the bottom fraction of the unit do not survive longer than 32 days. The survival time of RBCs has a linear correlation with the cells’ biomechanical and biochemical parameters and thus can be estimated using these parameters [35]. We therefore hypothesize that cells in the units stored for ≥ 14 days would not survive for more than 32 days based on the observation that the biomechanical and biochemical parameters are worse than those of the old RBCs in Unit 4. After 14 days of storage, even the RBCs that were originally in the Y fraction of a fresh RBC unit would have a survival time less than 46 (14+32) days. Therefore, the cells which have been stored for 21 days will become senescent soon and may not survive for more than one additional week.

The accelerated and/or aberrant physiological ageing of RBCs *in* *vitro* in CPDA-1 preservation solution is attributed to the acidic nature of the solution. As shown in Table 1, the pH value of the preservation solution decreased to 6.92 on the 14th day and then abruptly decreased to 6.53 on the 21st day due to the accumulation of lactic acid. These results are consistent with the findings previously reported [48], [49]. This pH decrease in the preservation solution can induce the loss of sialic acid from their membrane, which results in a decrease in charge. Decreases in cell surface charge, Na^+^/K^+^-ATPase activity, and 2, 3-DPG levels would induce structural changes of band 3 protein and lead to a collinear decrease of membrane deformability as well as morphological changes [35], [50]–[52]. These observations could explain why storage lesion develops quickly in the blood-banked RBCs. Consequently, the cells become senescent and do not have adequate deformability and oxygen carrying ability suitable for transfusion when they have been stored for 21 days.

Taken together, our data suggest that transfusions should use RBC units stored for ≤ 14 days. However, the mean storage time of RBC units before transfusion in some countries, such as China and the United States, is currently 17 to 19 days [53]. Consequently, there is an urgent requirement to improve the current storage conditions and preservation methods of banked blood and develop new techniques to retain the structural and functional properties of red cells for longer periods of time.

In summary, this paper presents for the first time the cell number as well as the biomechanical and biochemical parameters of RBCs in different age fractions of standard leuko-reduced CPDA-1 blood banked RBC units stored for various time periods. We demonstrated using substantive experimental evidence that the number of young, middle-aged (M1 and M2), and old cells progressively decline as a function of storage time. We also provided data showing how the RBCs of different cell ages have an increased ageing process and undergo cumulative structural and functional changes in the standard preservation conditions of blood bank during storage. Therefore, we can predict the RBCs’ fate after transfusion and evaluate the risk of transfusing the RBC units stored for different periods of time into circulation. We found that the RBC units stored for ≤ 14 days can be fractionated into four subpopulations of different ages; the total cell number in 1 mL of the RBC suspension after Percoll centrifugation was approximately 4.17×10^9^. The ratio of young to old cells decreased with longer storage periods from 1.33 on the 4^th^ day to 0.75 on the 14^th^ day. However, units stored for 21 days only exhibited three lower fractions that were equivalent to the M1, M2, and O fractions in Unit 14 and included many dead cell membrane fragments and microparticles as well as free hemoglobin, but not the Y fraction. The total cell number in 1 mL of cell suspension of the unit decreased to 3.2×10^9^, indicating that approximately 23% of the RBCs had been lost and the remaining cells were senescent. The evident regressive changes impacting the stored RBCs not only involve the cell phenotype, but also reflect a decline in the cell’s surface charge and deformability (Kc) as well as their Na^+^/K^+^-ATPase activity and 2, 3-DPG level. These changes are most likely induced by the acidification of the suspension pH with a longer storage time. This result could explain why less than 70% of the RBCs stored for a long period of time were alive 24 h post-transfusion. Since harmful membrane fragments, microparticles, and free hemoglobin were present in Unit 21, transfusion of the unit would not likely help patients and may induce adverse effects, such as in patients in intensive care [4], [12], [54]–[57] as well as those undergoing cardiac interventions [6], [13], [16], [58], [59], colorectal surgery [15], [60], and those with multiple trauma [14], [17], [18], [61]. We hope that our findings can help to explain the reason for these adverse effects as well as promote the development of novel blood storage systems and methods to prevent the decline of the RBC suspension pH and regressive changes in cell properties.
